# Corn Harvester Bearing Fault Diagnosis Based on ABC-VMD and Optimized EfficientNet

**DOI:** 10.3390/e25091273

**Published:** 2023-08-29

**Authors:** Zhiyuan Liu, Wenlei Sun, Saike Chang, Kezhan Zhang, Yinjun Ba, Renben Jiang

**Affiliations:** School of Mechanical Engineering, Xinjiang University, Urumqi 830047, China; 107552103797@stu.xju.edu.cn (Z.L.); 107552103901@stu.xju.edu.cn (S.C.); 107552103880@stu.xju.edu.cn (K.Z.); 107552101356@stu.xju.edu.cn (Y.B.); 107552103936@stu.xju.edu.cn (R.J.)

**Keywords:** corn harvester, fault diagnosis, artificial bee colony, variational mode decomposition, evaluation function, optimization of EfficientNet

## Abstract

The extraction of the optimal mode of the bearing signal in the drive system of a corn harvester is a challenging task. In addition, the accuracy and robustness of the fault diagnosis model are low. Therefore, this paper proposes a fault diagnosis method that uses the optimal mode component as the input feature. The vibration signal is first decomposed by variational mode decomposition (VMD) based on the optimal parameters searched by the artificial bee colony (ABC). Moreover, the key components are screened using an evaluation function that is a fusion of the arrangement entropy, the signal-to-noise ratio, and the power spectral density weighting. The Stockwell transform is then used to convert the filtered modal components into time–frequency images. Finally, the MBConv quantity and activation function of the EfficientNet network are optimized, and the time–frequency pictures are imported into the optimized network model for fault diagnosis. The comparative experiments show that the proposed method accurately extracts the optimal modal component and has a fault classification accuracy greater than 98%.

## 1. Introduction

As a critical component of corn harvesters, the significance of the bearing lies in its crucial role in guaranteeing the safety and dependability of the equipment operation [1]. In recent years, several technologies, such as big data and machine learning, have made significant progress in bearing fault diagnosis. In particular, big data technology can help process and analyze bearing operation data under different working conditions, and machine learning technology can efficiently mine the hidden features of these data to perform early detection and prediction of faults. Nevertheless, in the actual working environment, the vibration signal is often interfered with by many irrelevant signals due to the intricate operational conditions and the presence of noise interference in the equipment, which diminishes the precision of fault diagnosis. Therefore, it is of great significance to propose a bearing fault diagnosis method in complex environments to ensure the safe operation of corn harvesters [2].

The recent studies on bearing fault diagnosis focused on signal extraction and processing. The parameter selection of the VMD method significantly affects the decomposition results, which limits its application range in bearing fault diagnosis. Wang et al. [3] used the Tennessee Whisker search algorithm to optimize the search for the optimal parameter combinations of the VMD. The fitness function was defined as the inverse of the craggy value of each intrinsic modal function. Liang [4] enhanced the variational mode decomposition (VMD) method using an improved particle swarm optimization (IPSO) algorithm for the efficient extraction of fault features from non-stationary signals. Ye [5] introduced multiscale permutation entropy (MPE) in VMD to construct multidimensional feature vectors for fault classification. Although this method can optimize the VMD parameters, it is challenging to converge in some cases. JC et al. [6] used the genetic variant particle swarm algorithm for parameter optimization. They combined it with sample entropy for fault diagnosis, which improved the diagnostic accuracy to a certain extent but still had limitations when dealing with non-smooth signals. Addressing the issue of extracting fault features from nonlinear and non-stationary vibration signals of rolling bearings, this study aims to develop an effective approach that results in a low diagnosis and recognition rate. Liang [7] proposed an improved variational mode decomposition (VMD) and multi-feature feature extraction method based on the multi-island genetic algorithm (MIGA). Li [8] proposed a rolling bearing fault diagnosis method based on the variational mode decomposition-fractional Fourier transform (VMD-FRFT) to deal with the problem of over-decomposition in VMD. Li [9] used modified ensemble empirical mode decomposition (MEEMD) as a feature extraction technique. The latter mitigates the influence of the noise observed in ensemble empirical mode decomposition (EEMD). However, additional post-processing is necessary to diminish the presence of spurious components.

Bai et al. [10] proposed a method for the fault diagnosis of rolling bearings, which is based on the combination of the multi-channel convolutional neural network (MCNN) and the multiscale clipping fusion (MSCF) data enhancement technique. Niu et al. [11] proposed an optimized adaptive PReLU-DBN method for the identification of bearing faults. Huo [12] introduced an adaptive dimension-transformed convolutional neural network (ADC-CNN), which is able to dynamically convert one-dimensional vibration signals into two-dimensional matrices. This allows them to be efficiently processed by 2D-CNN for feature learning, thereby harnessing the strengths of CNN in extracting two-dimensional data features. Many classification models are currently used, such as EfficientNet, GoogleNet, and VGG, which are the mainstream models. Given the long training time and low classification accuracy of the original network, many experts and researchers have successively improved the model structure and used other algorithms to optimize the original model. Ding [13] proposed a reparameterized VGG (RepVGG)-based method to enhance the precision of bearing fault diagnosis. Wang [14] developed an improved 1D-CNN model based on VGG-16, which can input decomposed signals from different channels into separate convolutional blocks in the model and fuse them into the fusion layer. Gu [15] proposed a cosine similarity-based self-attentive Wasserstein generative adversarial network with gradient penalty (CSWGAN-GP) for bearing fault diagnosis under unbalanced conditions. Nijaguna [16] introduced ResNet50 and VGG16 to enrich the extracted features. Zhang [17] proposed an AM-ResNet model based on the convolution layer composition of addition and multiplication, which solved the problem of high energy consumption of the traditional ResNet. Gai [18] proposed a parameter-optimized deep belief network (DBN) to optimize the diagnosis difficulty due to the highly similar signals in fault features. Gang [19] combined neural network search techniques to balance the depth, width, and resolution of the network according to a specific ratio while balancing the speed and accuracy and optimizing the activation function of EfficientNet [19].

With the increase in the application of neural network models in fault diagnosis, the large scale of network parameters often leads to overfitting in the training process. Therefore, it is necessary to adopt a lightweight model to perform fault diagnosis with high generalization ability.

In summary, although VMD has improved the signal decomposition, it still has some problems, such as the number of decomposition layers and the penalty factor, which is challenging to select accurately. The EfficientNet classification model uses deep scalable convolutional blocks and lightweight feature extractors to provide the network with a strong representation with few parameters. However, the convergence process and classification accuracy of the model still have much room for improvement.

To this end, this paper proposes a bearing fault diagnosis method based on ABC-ANC and optimizes the EfficientNet. The main contributions of this study are summarized as follows.

(1) The artificial bee colony (ABC) algorithm is used to optimize the decomposition number K and the variational mode decomposition penalty factor. The permutation entropy, power spectral density, and signal-to-noise ratio are used to construct an evaluation function in order to select the best decomposition signal.

(2) The EfficientNet model optimized for the count of MBConv modules and activation function is proposed to improve the stability of the training process and the classification accuracy of the model.

## 2. Methodology

### 2.1. Variational Modal Decomposition (VMD) and Artificial Bee Colony Optimization

The VMD non-recursively decomposes a real-valued signal *x*(*t*) into k amplitude-FM sub-signals. To determine *u_k_* and *ω_k_*, the VMD can be written as a constrained variational problem described by the following equations:

(1) $$\underset{\left\{u_{k}\},\{\omega_{k}\right\}}{\min}\left.{\left\{\sum_{k}\left\|\left.\partial t\left[\left(\delta (t)+\frac{j}{\pi t}\right)*u_{k}(t)\right]e^{-j\omega_{k}t}\right\|\right.\right.}_{2}^{2}\right\}$$

(2) $$S.t.\begin{array}{cc} & \sum_{k}u_{k}=x \end{array}$$

The constrained variational problem is solved using the quadratic penalty term *α* and the Lagrange multiplier *λ*. The augmented Lagrange quantity function is given by:

(3) $$L(\{u_{k}\},\{\omega_{k}\},\lambda )=\alpha \sum_{k}{\left\|\left.\partial t\left[\left(\delta (t)+\frac{j}{\pi t}\right)*u_{k}\right]e^{-j\omega_{k}t}\right\|\right.}_{2}^{2}+{\left\|x(t)-\sum_{k}u_{k}(t)\right\|}_{2}^{2}+\langle \lambda (t),x(t)-\sum_{k}u_{k}(t)\rangle$$

where *α* is the equilibrium parameter of the data fidelity constraint.

The solution of the equation consists of updating each mode function and neutral frequency using the alternating direction multiplier method. The KTH amplitude-FM sub-signal obtained from the spectral domain solution is written as:

(4) $${\hat{u}}_{k}^{n+1}(\omega )=\left(\hat{x}(\omega )-\sum_{i\neq k}{\hat{u}}_{i}(\omega )+\frac{\lambda (\omega )}{2}\right)\frac{1}{1+2\alpha {\left(\omega -\omega_{k}\right)}^{2}}$$

The central frequency of each eigenmode component is expressed as [20]:

(5) $$\omega_{k}^{n+1}=\frac{\int_{0}^{\infty }\omega {\left|{\hat{u}}_{k}(\omega )\right|}^{2}d\omega }{\int_{0}^{\infty }{\left|{\hat{u}}_{k}(\omega )\right|}^{2}d\omega }$$

The ABC algorithm should first initialize the colony. That is, the SN d-dimensional initial solution *x*_i_ (i = 1, 2,…, SN) is randomly generated as:

(6) $$x_{i}=lb+\phi_{i}(ub-lb)$$

where SN is the population size, *lb* denotes the lower bound of the target space, *ub* represents the upper bound, *φi* represents a vector consisting of random values uniformly distributed between [0, 1] and *x_i_* = (*x_i_*_1_, *x_i_*_2_,…, *x_id_*)^T^.

Note that after initialization, the three bees search cyclically until reaching the maximum number of iterations. In the search phase, new food sources are randomly selected by the bees as follows:

(7) $$x_{ij}^{\prime }=x_{ij}+\phi_{ij}(x_{ij}-x_{kj})$$

where *x_ij_*(*j* = 1, 2, … d) is the JTH element of the *i*th solution, *j* and *k* are randomly selected within their value ranges.

According to Equation (7), the employed bee randomly selects a new food source, which fitness is then calculated as:

(8) $$fit_{i}=\left\{\begin{array}{l} 1/(1+f_{i})f_{i}\geq 0\\ 1+abs(f_{i})f_{i}<0 \end{array}\right.$$

The employed bee compares the fit value of the new scheme with the original scheme and chooses the best scheme. After the search, the employed bee gives the onlooker bee the latest information about food sources. The selection probability of each food source is then calculated according to Equation (9), and the watching bees select their food source scheme based on a roulette wheel style selection scheme.

(9) $$prob_{i}=fit/\sum_{i=1}^{SN}fit_{i}$$

During the bystander bee search phase, a random value is generated in the range of [0, 1]. If the random value is less than the probabilistic value, the relevant onlooker bee will find a new solution based on Equation (7). The onlooker bee then chooses a better solution, just like the employed bee. If the solution is not updated after a specified number, in an iteration known as limiting, in which employed bees give up their food source and become scout bees. This prevents the ABC from falling into a locally optimal solution, which is then randomized by the scout bee to choose a new food source. The traditional VMD decomposition usually leads to the problem of over-decomposition due to the improper choice of the decomposition mode (K) and penalty factor, which results in the deterioration of the input quality of the fault diagnosis model. Therefore, the artificial bee colony algorithm is used to optimize the selection of K and penalty factors in order to enhance the effectiveness and precision of the VMD decomposition.

**Figure 1:**
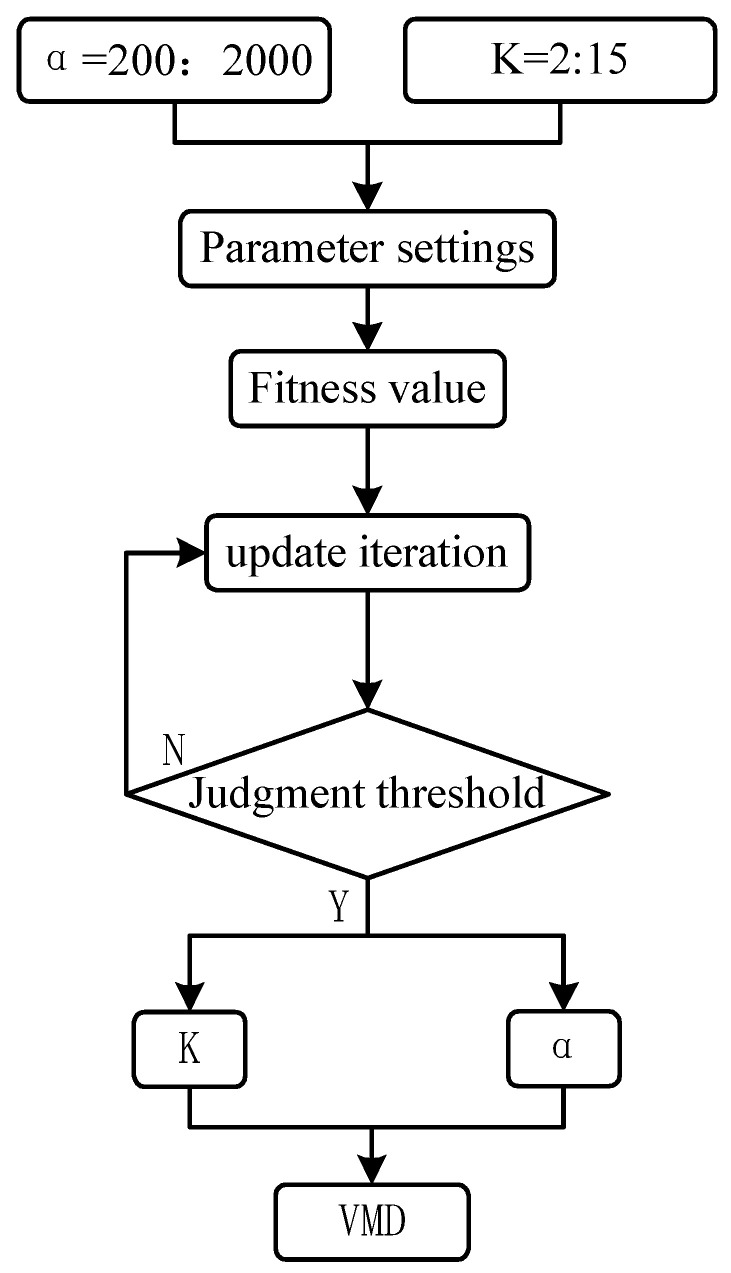
Flowchart of the variational modal decomposition for artificial bee colony optimization.

Figure 1 shows the specific process of optimizing variational modal decomposition by artificial bee colony, the overall process is summarized as follows.

Step 1: The parameter boundaries are determined, and the boundaries of K and α are initialized as [2, 15] and [200, 2000], respectively [21].

Step 2: The parameters of the artificial colony algorithm, including the number of food sources (SN), the ABC parameters (e.g., the proportion of employed bees and scout bees), the maximum number of iterations, and the limit value, are set. K and α are then randomly searched to decompose the input signal VMD, while the fitness value of the bee number of the ABC is calculated in each iteration.

Step 3: For the iteration termination conditions, a maximum number of iterations is set, and the best fitness value over several consecutive iterations below a set threshold is improved. If the iteration termination condition has not been met, the iterative process is repeated by updating the food sources K and α.

Step 4: The optimal K and α values based on the best fitness value are determined, and the original signal is decomposed by VMD using the optimal parameters.

### 2.2. Optimization of EfficientNet

In general, the bearing fault dataset cannot reach the million-level dataset required by EfficientNet to achieve the best classification effect. Too many MBConv modules in EfficientNet will result in high model complexity and computational workload. Therefore, this study adjusts and reduces the number of MBConv modules [22]. Adjustments were initially performed to the number of MBConv modules and the internal structure of the original network. More precisely, the 15 MBConv modules in the original network were reduced to 6. This modification aimed to minimize the complexity of the model and mitigate the risk of overfitting on smaller datasets. The enhanced version of EfficientNet incorporates only two convolutions with an extension factor of 6 in the fourth MBConv stage while employing only one convolution in the other stages.

**Figure 2:**
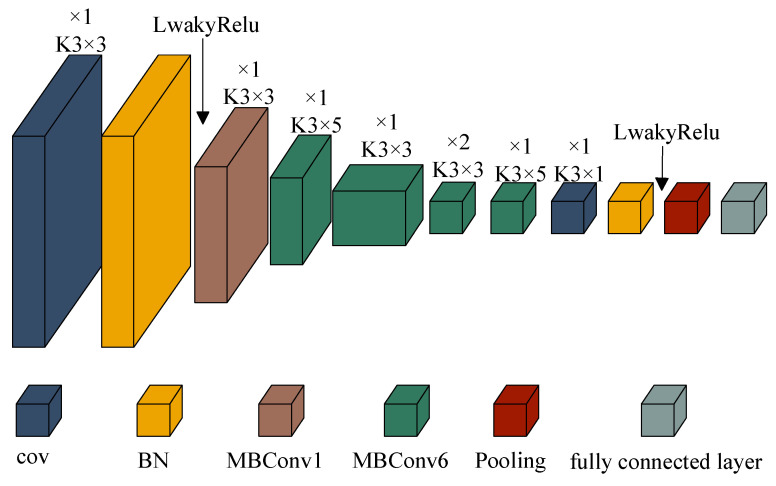
Optimized EfficientNet.

The primary role of the activation function is to provide nonlinear expressivity to the neural network. The first-order derivative of the LeakyRelu activation function is constant, while the second and first-order derivatives of the Swish activation function are not constants, and the derivation process is more complex [22]. Although the intricate derivation process does not contribute to the enhancement of the neural network performance, it merely escalates the training time and computational burden of the network. In this study, the improved EfficientNet employs the LeakyReLU activation function instead of the Swish activation function. The detailed architecture of EfficientNet is illustrated in Figure 2, where K represents the size of each convolutional kernel.

## 3. Bearing Fault Diagnosis Process and Model

**Figure 3:**
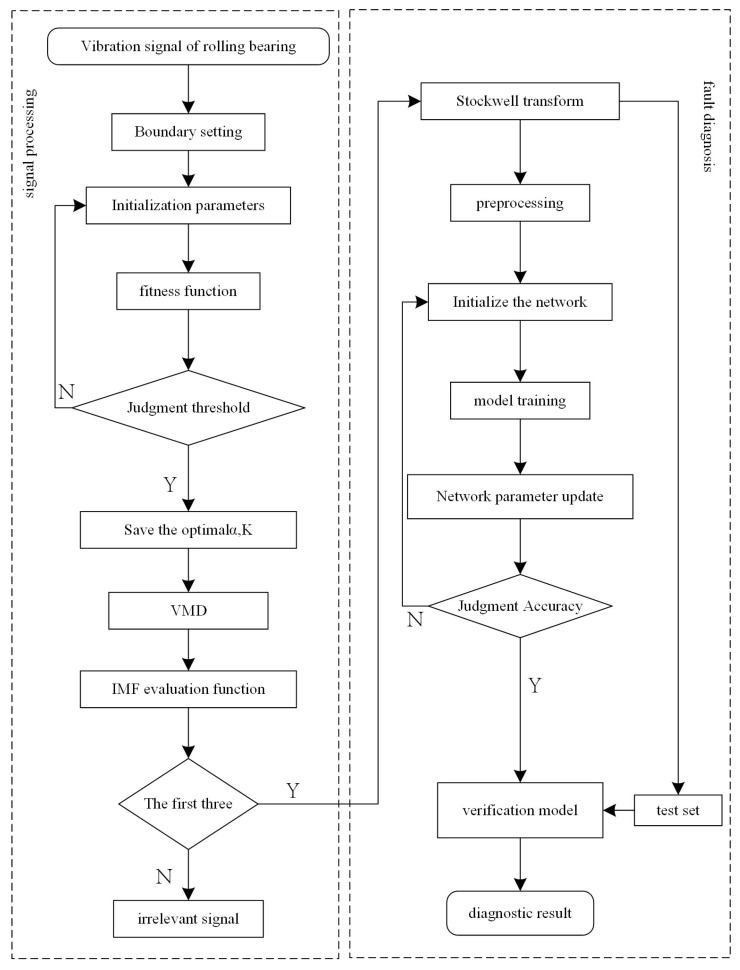
Rolling bearing fault diagnosis process.

In this section, the precise diagnostic procedure shown in Figure 3 is detailed.

The bearing fault diagnosis classification model based on the ABC-VMD and optimized EfficientNet mainly comprises five steps.

Step 1: The vibration signals of bearings under normal operation, ball fault, inner ring fault, and outer ring fault state are collected and optimized through the test bench, and vibration characteristics under different fault states are obtained. The irrelevant signals are eliminated by signal filtering using several techniques, such as thresholding to filter out the data that are not within the expected range and by replacement for irrelevant or missing data using interpolation or other methods.

Step 2: ABC-VMD is used to find the optimal penalty factor and decomposition number. The best parameters for the modal decomposition of the vibration signal collected in Step 1 are determined.

Step 3: The permutation entropy [23], power spectral density [24], and signal-to-noise ratio [25] are calculated for each signal component. Note that the arrangement entropy measures the signal complexity, and the signal-to-noise ratio assesses the signal quality. These three metrics are fused to construct an evaluation function in order to determine the valuable components of the signal, and the top three most significant components of the evaluation function are selected.

Step 4: The selected signal components are converted to time–frequency images using the Stockwell transform [26]. The latter is a time–frequency analysis method that provides good resolution in the time and frequency domains, capturing localized signal features to identify better types of bearing faults.

Step 5: The generated time–frequency images are fed into the optimized EfficientNet model for training and validation.

The use of VMD as the signal processing means of the proposed method has the following advantages [27].

(1) Adaptive decomposition: In contrast to the traditional fixed-base decomposition methods, VMD can adaptively select the decomposition mode to better match the characteristics of the signal.

(2) Nonlinear and non-stationary signal analysis: VMD is suitable for analyzing nonlinear and non-stationary signals. It can adapt to the local characteristics of the signal, which allows it to provide a more precise analysis.

(3) No prior information required: In contrast to the other methods, VMD does not require prior information about the signal or noise.

(4) Sparsity: VMD can usually produce relatively sparse solutions, with each mode capturing only one specific signal component. This helps to better understand and visualize the structure of the signal.

(5) Fewer pseudo components and mode mixing: VMD tries to reduce the generation of spurious components and the mixing between different modes, which provides a cleaner decomposition.

(6) Ability of real-time analysis: Due to its mathematical properties, VMD can be quickly executed, which makes it suitable for real-time or near-real-time signal analysis applications.

(7) Robustness: VMD has a certain robustness and resistance to noise and other disturbances.

However, the traditional VMD decomposition usually leads to problems such as over-decomposition due to the improper selection of the decomposition mode number (K) and penalty factor, which results in decreasing the input quality of the fault diagnosis model. Therefore, although the following problems are faced when dealing with large-scale datasets [28], artificial bee colonies are used to search for the optimal parameters.

(1) Local searchability: ABC has a low local search ability, which leads to getting stuck when approaching the global optimal solution.

(2) Adaptability to complex problems: The ABC algorithm may need to perform better for some very complex or high-dimensional optimization problems.

(3) Parameter tuning: Although fewer parameters exist, their choice may affect the performance of the algorithm. For different problems, the parameters may require to be accurately tuned.

(4) Possibility of falling into local optimum: In some cases, especially in complex multimodal function optimization problems, ABC may fall into the local optimum solution.

Therefore, the artificial bee colony algorithm has higher performance on the small dataset of bearings collected in this paper. More precisely, it has the following characteristics.

(1) Global search ability: ABC can perform a global search in the solution space, and it can find the global optimal solution.

(2) Robustness: Compared with many other optimization methods, ABC is less sensitive to the selection of initial parameters, and it has a higher robustness.

(3) Ease of implementation: The rules of ABC are straightforward, and it can be easily implemented.

(4) Existence of a small number of control parameters: ABC only requires a small number of control parameters that can be easily adjusted and optimized.

(5) Parallelism: The natural parallel nature of the algorithm allows it to be effectively implemented in a parallel computing environment.

The Stockwell transform in step 4 can provide a joint representation of the time and frequency of the signal. It then preserves the phase information of the signal, which allows a more accurate time–frequency and multi-resolution analysis. It has higher time resolution at low frequencies and higher frequency resolution at high frequencies [26]. Therefore, the extracted bearing characteristic signal is input into EfficientNet after Stockwell transformation.

## 4. Experimental Analysis

### 4.1. Case Western Reserve Experimental Dataset Validation

#### 4.1.1. Dataset Introduction

**Figure 4:**
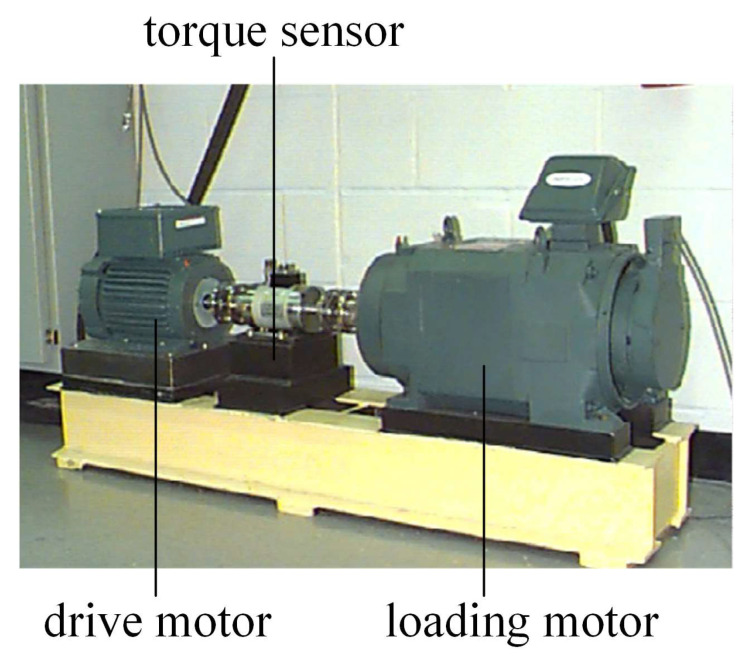
CWRU Bearing Test Stand.

The bearing failure dataset used in this paper is derived from the bearing dataset provided by Case Western Reserve University (CWRU). The experimental setup shown in Figure 4 contains a 1.5 kW drive motor, a torque sensor, and a loading motor. The sampling frequency is 12 kHz [29]. During the experiment, an accelerometer was installed at the fan end and the drive end bearing housings of the electric motor. These sensors were used to collect the vibration signals from the drive-end bearing, while the data were recorded using a data acquisition device.

In the experiment, the SKF6205 bearing type was divided into four states: normal, ball defects, inner ring failure, and outer ring failure. The failure under the load was 0, 1, 2, and 3 hp, where the damage degree of each failure diameter size was 0.007, 0.014, and 0.021 inches, leading to a total of 10 conditions of the bearing data. The dataset consists of 9660 samples, with 6720 and 2940 samples allocated to the training set and testing set, respectively. The details of the dataset are shown in Table 1.

#### 4.1.2. Construction of the Stockwell Time–Frequency Graph Sample Set

In order to determine the valuable signal components, a comprehensive evaluation function is established, which fully considers the permutation entropy, PSD mean, and signal-to-noise ratio. The evaluation function is given by:

Score=ω1×SNR+ω2×PSD mean−ω3×PE

where ω1, ω2, and ω3 are the weighting parameters.

The magnitude of change in each indicator is the basis for the selection of the weighting parameters. More precisely, the magnitude of the components of the permutation entropy transformation is relatively small, and thus, the components of the permutation entropy are set with smaller weights. The evaluation function is characterized by the combined permutation entropy, PSD mean, and signal-to-noise ratio in a weighted manner. The permutation entropy, PSD mean, and signal-to-noise ratio reflect the complexity, energy distribution, and intensity of the signal relative to the noise, respectively.

The process of parameter optimization based on ABC-VMD is summarized as follows.

(1) Initialization: The kurtosis is defined as the objective function for assessing the quality of the VMD, and the artificial bee colony is initialized. Each bee represents a possible solution (i.e., the values of ‘K’ and the penalty factor).

(2) Evaluation: For each solution in the bee colony, VMD decomposition is performed, and the obtained result is evaluated using the defined objective function.

(3) Selection: Based on the assessment of the objective function, the best solutions for the worker bees to continue their search are selected while leaving the rest as scout bees to explore new potential solutions.

(4) Search Optimization: The worker bees employ localized search to improve their solutions, while the scout bees use random search strategies to find entirely new solutions.

(5) Iterative Process: The aforementioned steps are repeated until certain stopping criteria are met, such as reaching a maximum number of iterations or the solution’s improvement falls below a specific threshold.

(6) Result Extraction: The optimal solution is extracted from the bee colony, identified by the best objective function value.

The final optimal component number (K) is set to 6. Table 2 shows the specific indicators of each component. In order to unify the data scale, the calculated permutation entropy (PE), signal-to-noise ratio (SNR), and mean power spectral density (PSD) are normalized.

The calculation process of the evaluation function based on the data in Table 2 is summarized as follows. The variation range of the PE, SNR, and PSD is first judged by the variance, which is 0.0002095, 0.99909, and 0.4028, respectively. It can be clearly seen that the SNR has the highest variation range, and thus, ω1 is set to 0.5, followed by the SNR, while PE has the lowest value. To ensure the weight balance, ω2 and ω3 are set to 0.3 and 0.2, respectively. Taking IMF3 as an example, the score is equal to 0.5 × 0.679 + 0.3 × (−0.445) − 0.5 × 0.565 ≈ 0.093, and the remaining signal scores are calculated according to the above process.

**Figure 5:**
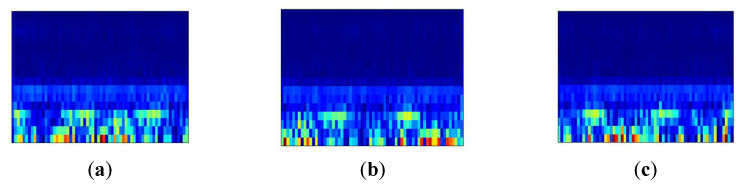
Stockwell transformed time–frequency waveform signal. (**a**) IMF1, (**b**) IMF2, (**c**) IMF3.

The magnitude of the IMF4 evaluation function is significantly reduced in Table 2. The score in this context represents the clarity of the features. More precisely, a higher score indicates fewer irrelevant signals. Therefore, to match the three-channel input layer of EfficientNet [22], the first three IMF components are selected for the Stockwell transform in order to generate the time–frequency image. In order to construct the time–frequency image sample set, the time–frequency images of the first three groups of IMF components are considered as three channels to form a multi-channel image, which better preserves the spatial relationship between the components and improves the recognition ability of the model. The time–frequency image sample set composed of these multi-channel images can be directly input into the model for bearing fault diagnosis. Figure 5 shows the time–frequency image generated using the first three IMF components.

#### 4.1.3. Comparison of Evaluation Functions

**Figure 6:**
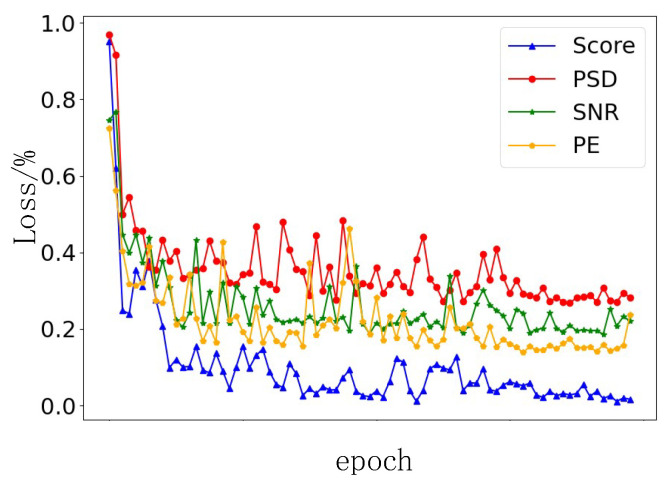
Comparison between the model losses for different evaluation functions.

In order to verify the outperformance of the evaluation function score, the PE, SNR, and PSD are compared with the evaluation function while considering the same inputs and the same training model to avoid the effect of random errors. The loss function of the signals extracted by different indicators in the network training process is shown in Figure 6.

It can be seen that in the first ten rounds of iterations, the four models have a downward trend. However, when the iteration period gradually increases, the gap between the signal extracted by the PE, SNR, and PSD and the evaluation function score becomes gradually noticeable, which indicates that the modal features extracted by the indicators in this paper are more conducive to model training. After the tenth iteration, the loss value of the evaluation function score is significantly lower than the other three, showing a good model training process.

#### 4.1.4. Comparison between Classification Models

**Figure 7:**
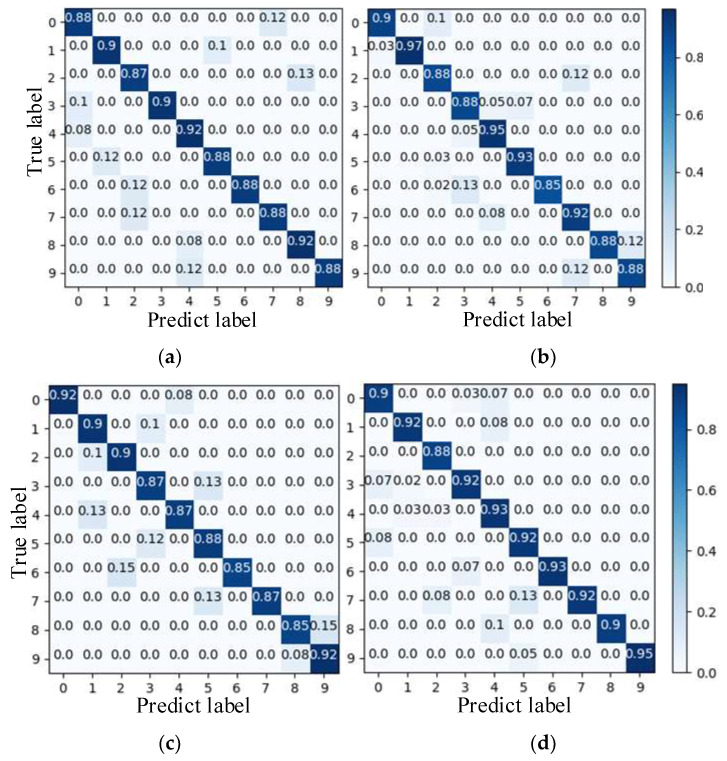

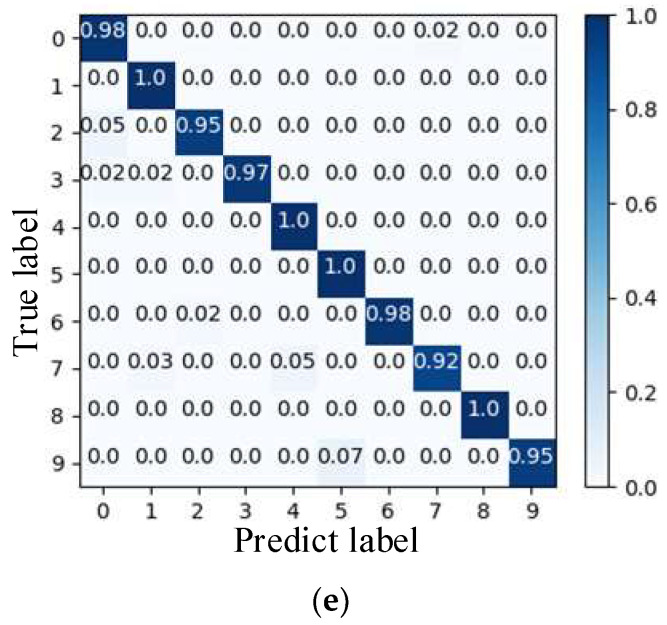
Comparison between the fault identification results obtained using different models. (**a**) VGGNet; (**b**) DenseNet; (**c**) ResNet; (**d**) EfficientNet; (**e**) optimized EfficientNet.

To further demonstrate the outperformance of the proposed method in terms of fault classification, a confusion matrix comparison is performed using EfficientNet and a high-parameter number network. The obtained results are shown in Figure 7, where the abscissa is the predicted label, and the ordinate is the actual label.

VGGNet [30], DenseNet [31], and ResNet have a larger parameter search space in the model training process due to the higher number of parameters, which makes the optimization and adjustment more challenging. It can be seen from Figure 7a–c that the average accuracy of bearing fault diagnosis is only 89%, 90.67%, and 88.33%. Figure 7d,e shows that the optimized EfficientNet network identifies up to 100% for some bearing faults with an average accuracy of 97.33%, while EfficientNet identifies only 91.67%.

**Figure 8:**
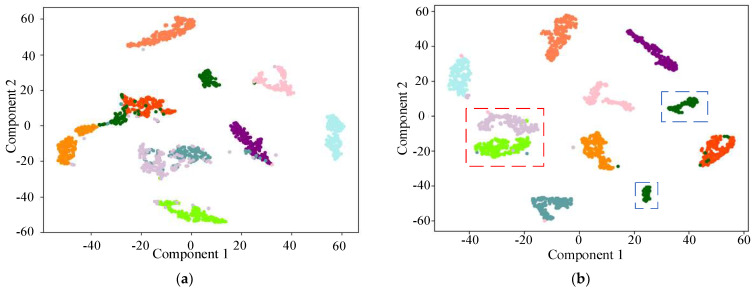

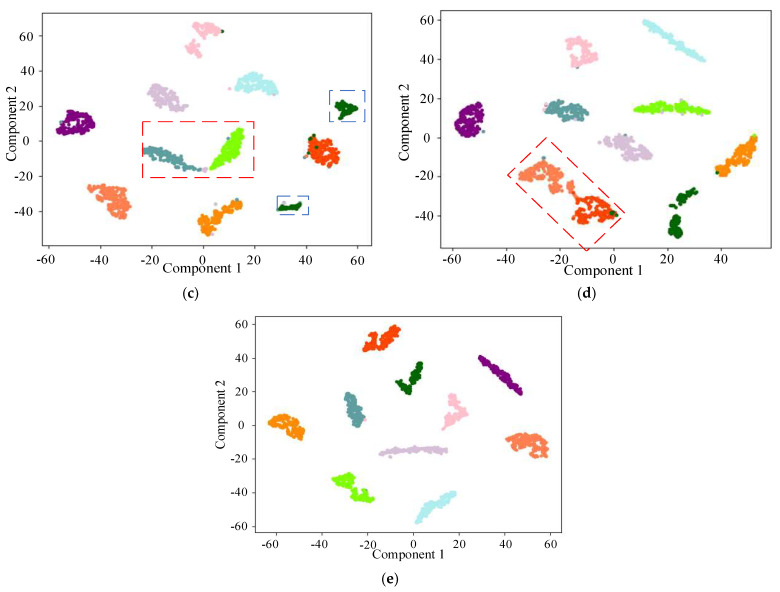
Comparison between the feature extraction visualization results obtained by different models. (**a**) VGGNet; (**b**) DenseNet; (**c**) ResNet; (**d**) EfficientNet; (**e**) optimized EfficientNet.

In order to more intuitively show the feature extraction ability of the proposed model, the t-SNE [32] dimension reduction visualization technology is used to visualize the results of the test set, as shown in Figure 8. It can be seen from Figure 8a that all the VGGNet fault classes are mixed and indistinguishable. Figure 8b–e shows the apparent modal aliasing phenomenon (red box part) and clustering failure phenomenon (blue box part) among different faults. However, the proposed fault diagnosis method does not show any overlapping areas, which indicates a good bearing fault diagnosis effect.

### 4.2. Test Verification of 4YZB-8B Self-Propelled Corn Harvester

#### 4.2.1. Comparison between High Parameter Models

**Figure 9:**
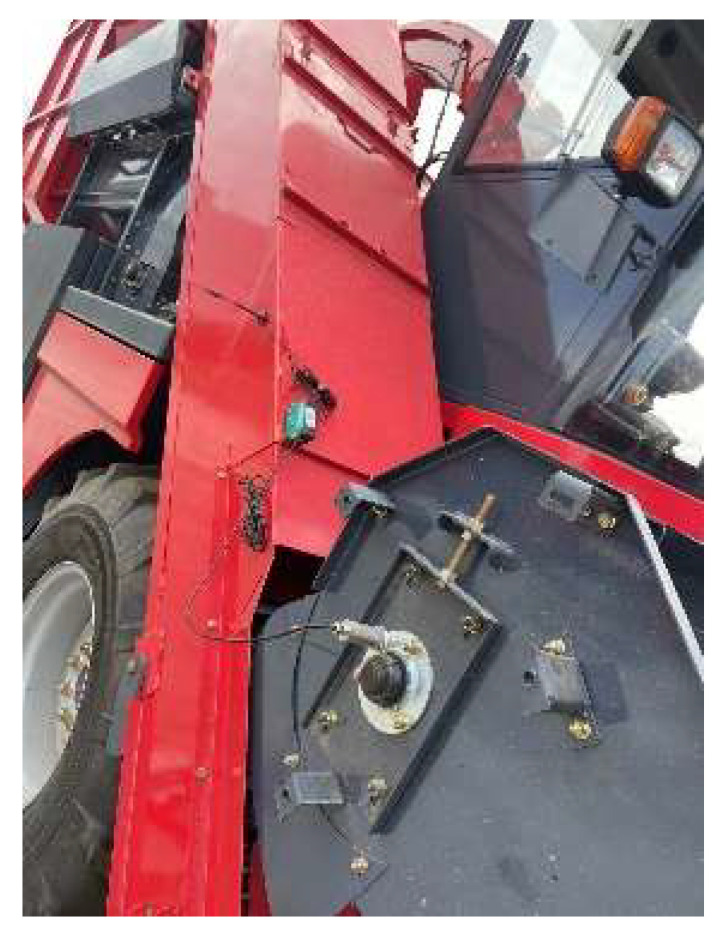
4YZB-8B self-propelled corn harvester.

In the actual working process, there are differences in the working environment of the bearings and the mode of operation, leading to deviations in the fault signals. The low robustness and generalization ability of the model affects the learning ability and the degree of fitting, which reduces its fault diagnosis accuracy. Therefore, the bearing fault signal of the bearing of the corn harvester under the working state is collected on the spot. Figure 9 presents a 4YZB-8B self-propelled corn harvester, simulating vibration signals under the normal bearing, inner ring, single point, and multi-point fault state.

**Figure 10:**
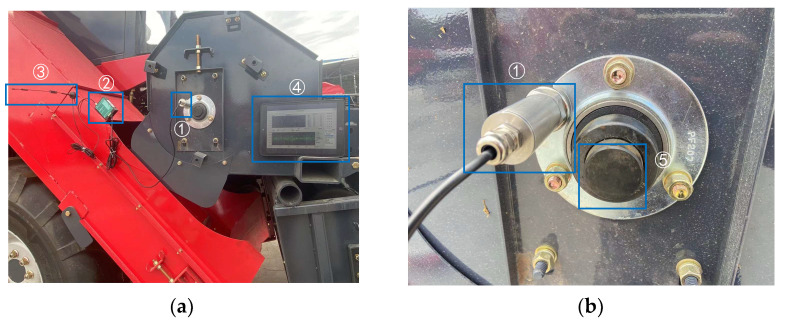
The acquisition device. (**a**) Overall view; (**b**) Local view.

The acquisition device is shown in Figure 10a, and the location of the measuring points is shown in Figure 10b. The wireless sensor model PR-3001-W23-N01-CX is manufactured by Shandong Sean Electronic Technology. Its vibration speed accuracy is ±1.5% of full scale (FS), and it was acquired in early 2023. The life expectancy of the device is determined by the default parameters and typically amounts to approximately eight years. A separate component, the data transmission device, carries the model number PR-300YM-4G and is configured to transmit data at 30-s intervals. The sampling frequency is 4 kHz. Note that ➄ is the transmission shaft. After vibration sensor ➀ collects the signal, it is transmitted to the tablet computer ➃ through the network data acquisition instrument ➁ and antenna ➂ using 4G technology.

The labels of the collected fault datasets are shown in Table 3.

**Figure 11:**
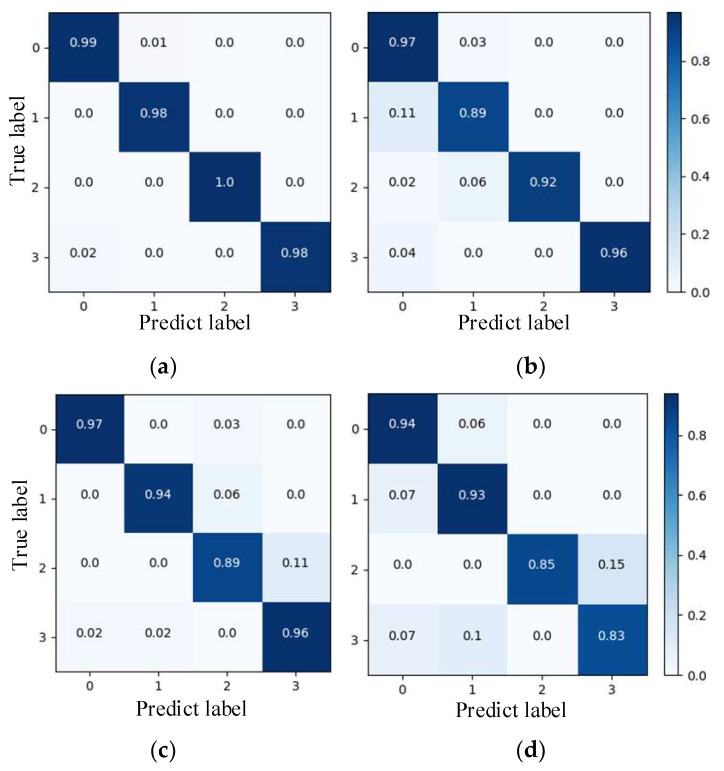
Confusion matrix for different models. (**a**) optimized EfficientNet; (**b**) EfficientNet; (**c**) DenseNet; (**d**) VGGNet.

In order to compare the recognition effects of the proposed model for different bearing datasets, the confusion matrix is introduced for EfficientNet, DenseNet, and VGGNet to verify the recognition accuracy of the training model in different fault samples in the test set. Figure 11 illustrates the recognition results for each model. It can be seen from Figure 11a that, in general, the proposed model outperforms the other four types in faulty bearing identification. More precisely, all its accuracy rates are greater than 98%. Figure 11b shows that the EfficientNet model has a misdiagnosis rate of up to 11% for inner-ring faults. Figure 11c shows that the DenseNet model performs the worst in identifying single-point faults in the outer ring, with an average recognition accuracy of only 89%. It can be observed from Figure 11d that the VGGNet model is less effective in identifying two types of bearings (single-point faults and repeated faults) on the outer ring, and it cannot provide reliable guidance for the judgment of bearing faults.

**Figure 12:**
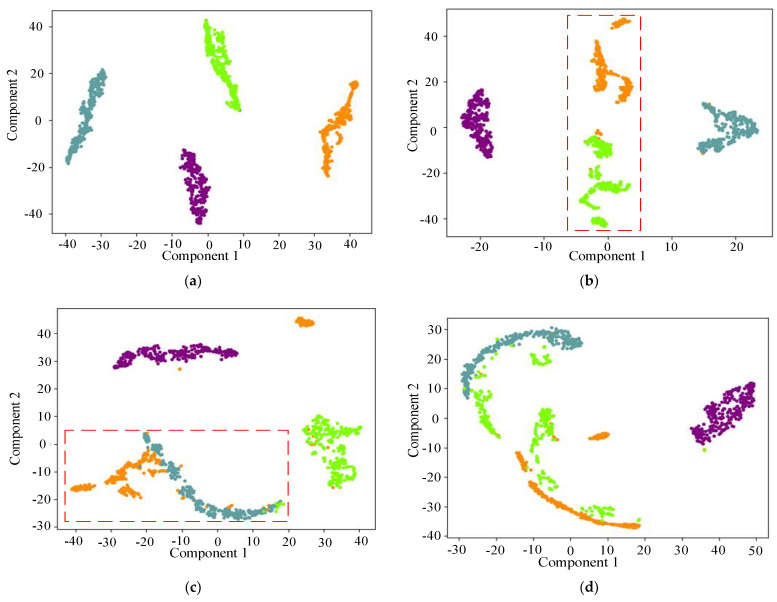
Distribution of t−SNE on the fully connected layer for different models. (**a**) optimized EfficientNet; (**b**) EfficientNet; (**c**) DenseNet; (**d**) ResNet.

In order to intuitively compare the classification abilities of the six models, t-SNE [32] is used to visualize the high-dimensional features. The extracted high-dimensional features from the six models are projected onto the 2D plane, as shown in Figure 12.

It can be seen from Figure 12 that the optimized EfficientNet alignment strategy outperforms the other methods, while EfficientNet and DenseNet have aliasing and large distances in the same category to varying degrees (red box part). On the contrary, the proposed model does not have these shortcomings. It leads to a small distance of feature distribution within the identical category and a significant gap between distinct classes, which is more conducive to fault classification.

#### 4.2.2. Comparison between Existing State-of-the-Art Studies

To further demonstrate the high accuracy of the proposed method, it is compared with existing state-of-the-art approaches. The obtained results are shown in Table 4.

(1) The approach presented in [33] is a bearing fault detection method based on ResNetV2. The collected 1D bearing data are converted to 2D, solving the workload that manual feature extraction increases. For the bearing dataset of the 4YZB-8B self-propelled corn harvester, although the fault recognition rate of the method is higher in the second and third tests (reaching 98.6% and 97.2%, respectively), the fault recognition rate of the first test is only 86.2%. The results show that the low robustness of the bearing fault diagnosis of the 4YZB-8B self-propelled corn harvester based on ResNetV2 is attributed to the overfitting caused by the excessive number of covariates of the model, which varies the fault recognition accuracy.

(2) The authors of [34] introduced various deep learning algorithms for the prognostics and health management (PHM) of rotating machinery, including the restricted Boltzmann machine (RBM), deep belief network (DBN), deep Boltzmann machine (DBM), auto-encoder (AE), convolutional neural network (CNN), and recurrent neural network (RNN). For the three fault identification tests, the overall performance of the six models on the bearing dataset of the 4YZB-8B self-propelled corn harvester is poor. This is due to the fact that all these models require a large amount of data and computational resources for training, which leads to difficulties in model convergence and low generalization ability in the case of insufficient training samples.

(3) The method presented in [35] uses a generative adversarial network (GAN) to solve the problem of severe lack caused by equipment. When identifying the bearing dataset of the 4YZB-8B self-propelled corn harvester, although the accuracy rate is as high as 97.2% in the first test, it is not stable, and it drops by 8% in the second identification.

The proposed optimization of EfficientNet not only retains the advantages of the original model but also reduces the complexity and avoids overfitting, leading to an average accuracy rate of 98.7%.

**Table 4:** Comparison between the proposed method and existing state-of-the-art studies.

Test	Method Presented in [33]	RBM	DBN	DBM	AE	CNN	RNN	Method Presented in [35]	Proposed Method
First test	86.2%	85.2%	94.2%	95.2%	93.2%	80.4%	90.2%	97.2%	98.7%
Second test	98.6%	79.2%	93.7%	93.2%	94.6%	78.6%	88.3%	89.2%	98.3%
Third test	97.2%	78.6%	92.1%	94.6%	91.7%	72.1%	87.3%	96.8%	99.1%

## 5. Conclusions

Fault characteristic signals are often submerged due to the complex working environment of corn harvester bearings. The accuracy and stability of the fault classification model depend on the characteristic signal processing and the convergence ability of the diagnosis model.

The conclusions of this study are summarized as follows.

(1) A fault signal decomposition method based on ABC-VMD is proposed. It relies on the ABC algorithm to search for the optimal decomposition number and penalty factor in order to improve the accuracy of fault feature extraction.

(2) By combining the complexity of the signal, the ratio between the signal quality and the noise level, and the energy distribution in the frequency domain, the evaluation function based on the permutation entropy, signal-to-noise ratio, and power spectral density is constructed, which can fully reflect the fault signal characteristics.

(3) An optimization approach for EfficientNet is proposed to solve the problem of high complexity and computational workload of the high parametric quantity models. The convergence ability of the model is effectively improved. The number of MBConv modules is optimized. In addition, LeakyRelu is used to reduce the calculation workload and optimize the iterative process of the fault classification model.

Although the proposed method outperforms other existing models, after optimizing EfficientNet, the number of modules is reduced, which is mainly suitable for small datasets and can lead to model underfitting in the case of large amounts of data. Therefore, in future work, we aim to avoid the impact of different sample volumes on the recognition accuracy of EfficientNet.

## Figures and Tables

**Table 1:** Bearing datasets.

Fault	Fault Diameter (inch)	Label	Count of Samples	Training Set	Testing Set
normal	0	0	9660	6720	2940
inner ring failure	0.007	1			
	0.014	2			
	0.021	3			
outer ring failure	0.007	4			
	0.014	5			
	0.021	6			
ball defects	0.007	7			
	0.014	8			
	0.021	9			

**Table 2:** IMF component parameters.

IMF	PE	SNR	PSD Mean	Score
IMF1	0.557	1.244	2.207	1.1727
IMF2	0.521	0.687	−0.100	0.2093
IMF3	0.565	0.679	−0.445	0.093
IMF4	0.540	−0.205	−0.534	−0.3707
IMF5	0.536	−0.664	−0.559	−0.6069
IMF6	0.537	−1.741	−0.566	−1.1477

**Table 3:** 4YZB-8B dataset.

Fault		Label	Count of Samples	Training Set	Testing Set
Normal		0	7700	5600	2100
inner ring failure		1			
outer ring failure	single point fault	2			
	multi-point fault state	3			

## Data Availability

The public dataset from CWRU can be found at https://engineering.case.edu/bearingdatacenter (accessed on 1 March 2023). The signals collected from the 4YZB-8B self-propelled corn harvester are owned by the author’s institution and are not allowed to be used in a public way.
